# Checking Agriculture’s Pulse: Field Pea (*Pisum Sativum* L.), Sustainability, and Phosphorus Use Efficiency

**DOI:** 10.3389/fpls.2019.01489

**Published:** 2019-11-15

**Authors:** Sarah E. Powers, Dil Thavarajah

**Affiliations:** Plant and Environmental Sciences, 270 Poole Agricultural Center, Clemson University, Clemson, SC, United States

**Keywords:** field pea, organic farming, phosphorus, pulse crop physiology, biofortification

## Abstract

Investigations regarding the incorporation of better sustainable production strategies into current agricultural-food systems are necessary to grow crops that reduce negative impacts on the environment yet will meet the production and nutritional demand of 10 billion people by 2050. The introduction of organic, alternative staple food crops, such as nutrient-dense field pea (*Pisum sativum* L.), to the everyday diet, may alleviate micronutrient malnutrition and incorporate more sustainable agriculture practices globally. Varieties are grown in organic systems currently yield less than conventionally produced foods, with less bioavailable nutrients, due to poor soil nutrient content. One of the most limiting nutrients for field pea is phosphorus (P) because this legume crop requires significant inputs for nodule formation. Therefore, P use efficiency (PUE) should be a breeding target for sustainable agriculture and biofortification efforts; the important role of the soil microbiome in nutrient acquisition should also be examined. The objectives of this review are to highlight the benefits of field pea for organic agriculture and human health, and discuss nutritional breeding strategies to increase field pea production in organic systems. Field pea and other pulse crops are underrepresented in agricultural research, yet are important crops for a sustainable future and better food systems. Furthermore, because field pea is consumed globally by both developed and at-risk populations, research efforts could help increase global health overall and combat micronutrient malnutrition.

## Introduction

The Green Revolution is indisputably one of the most critical feats in recent agricultural history, but what has it cost our soils, crops, and environment as a whole? One result of the focus on mass production in monocultural systems for prolonged periods is the over-application of fertilizers and pesticides, which is a prevalent issue associated with conventional farming methods (Ponisio et al., 2015). As a result, soil fertility and microbial biodiversity have decreased while rates of environmental pollution and greenhouse gas emissions continue to increase (Reganold et al., 1987; Amundson et al., 2015; Reganold and Wachter, 2016; Peoples et al., 2019). Organic agriculture offers a potential solution to these problems, as organic production relies on environmentally friendly practices to increase soil fertility. However, current varieties bred for conventional systems do not perform as well in organic soils, resulting in reduced yield and nutritional quality. The agriculture industry as a whole has also begun to deplete the natural mineral deposits on which crops depend, such as phosphate rock, which is a nonrenewable resource (van de Wiel et al., 2016). Organic and conventional agriculture both use phosphorus rock for around 90% of the phosphorus (P) found in fertilizers, feed, and other food additives; however, most P is subsequently lost from the food system due to mining and field practices (Cordell and White, 2014; Amundson et al., 2015). The P that is applied as a fertilizer is also often mismanaged; specifically, it is over-applied to fields, leading to a build-up of the element in the soil where it is inaccessible to plants due to its immobile nature and affinity to form insoluble complexes with other minerals (Vance et al., 2003; MacDonald et al., 2011). Experts cannot seem to agree on when phosphorus reserves will run out, with estimates between the years 2030 and 2100 (van de Wiel et al., 2016); regardless, agriculture must still address its current P problem for future crop production.

Phosphorus is vital to agriculture because it is required by all plants, being involved in seed germination, root growth, structure development, and numerous metabolic processes such as photosynthesis and nutrient formation (van de Wiel et al., 2016). Therefore, when P is limited in soils it negatively affects not only plant growth and yield but also the nutrient concentration and bioavailability in food crops, leading to micronutrient deficiencies or “hidden hunger” (Assuero et al., 2004; Welch and Graham, 2004; Rehman et al., 2018). Potential solutions to hidden hunger include: 1) biofortification to increase bioavailable micronutrients in staple crops through agronomic, plant breeding, and biotechnology efforts (Welch and Graham, 2004) and 2) diversifying staple crops to include cheaper, environmentally sustainable, and more nutrient-dense foods, such as field pea (*Pisum sativum L*.) and other pulse crops (Foyer et al., 2016).

Field pea is a member of the Leguminosae family, along with faba bean (*Vicia faba*), grass pea (*Lathyrus sativus*), white lupin (*Lupinus albus*), lentils (*Lenis culinaris*), mung bean (*Vigna radiata*), soybean (*Glycine max*), cow pea (*Vigna ungulicata*), and common bean (*Phaseolus vulgaris*) among others (Foyer et al., 2016). Additionally, Leguminosae consists of the subfamily Papilionideae which splits into two distinct clades of cultivated legumes: 1) Hologalegina, evolving 50 million years ago and 2) Phaseoloid, evolving 45 million years ago (Foyer et al., 2016). These clades evolved separately, as Hologalegina is comprised of all cool season legumes, such as field pea, lentil, faba bean, and grass pea, while Phaseoloids consists of warm-season legumes (pigeon pea, soybean, common bean, mung bean, and cowpea) (Foyer et al., 2016). Cool season legumes are critical to sustainable agriculture, as they are planted during winter, complementing the growing season of cereals, and providing essential nitrogen and other nutrients back to the soil.

Field pea is a critical economic and nutritive crop and is often called “poor man’s meat” due to its high protein, vitamin and mineral, and prebiotic carbohydrate content yet affordability for poorer consumers (Amarakoon et al., 2012). More specifically, field pea is naturally rich in iron and zinc and thus, could address two of the most common micronutrient deficiencies in the world (Amarakoon et al., 2012). Despite the potential for the higher consumption of field pea to help alleviate hidden hunger, little advancement has been made to increase production and yields have lagged behind those of cereals (Amarakoon et al., 2012). One of the main issues with field pea, and legumes, in general, is that they require much more P input than other crops due to their nodules, which require P for energy transformation (Vance et al., 2003); this presents an issue for sustainable agriculture.

### Field Pea Benefits Agriculture

Field pea is one of the oldest domesticated pulse crops, appearing in the Mediterranean between 7000 and 6000 BC and persisting in current agriculture (Helback and Hopf, 1959). Pulse crops are a category of legumes, with seeds specifically harvested at full maturity (FAO, 1994). Pulses are very beneficial to agriculture systems, achieving large success in sustainable agriculture systems through intercropping and crop rotations with cereals. Pulses are able to break disease and weed cycles associated with cereals, while replenishing nitrogen (N) in the soil through their ability to fix N from the atmosphere through their nodules and symbioses with rhizobia. Globally, 21 Mt of nitrogen is fixed by legumes, with 5–7 Mt returned to the soil by pulses, specifically, which saves U.S. farmers $8-12 billion in total (Foyer et al., 2016). In Australia, farmers reported a 30% increase in wheat after adding a legume rotation compared to monocropped wheat (Stagnari et al., 2017). Studies from Denmark also report nitrogen uptake of various crops increases between 23–59% after rotations with field pea and lupin (Stagnari et al., 2017). As N is another of the most limiting nutrients for cereal and crop production, this legume-mediated increase in nitrogen use efficiency offers a sustainable and cost-effective alternative to high input fertilizer regiments. Pulses also foster other beneficial properties for soil health, such as increased biodiversity, soil organic carbon (SOC) levels, and soil water retention, while decreasing greenhouse gas emissions (GHG) (Foyer et al., 2016; Stagnari et al., 2017; Peoples et al., 2019). Field pea has the most positive effect on SOC by improving humus levels and supplying organic C as a result of bacterial nitrogen fixation (Stagnari et al., 2017).

**Figure 1:**
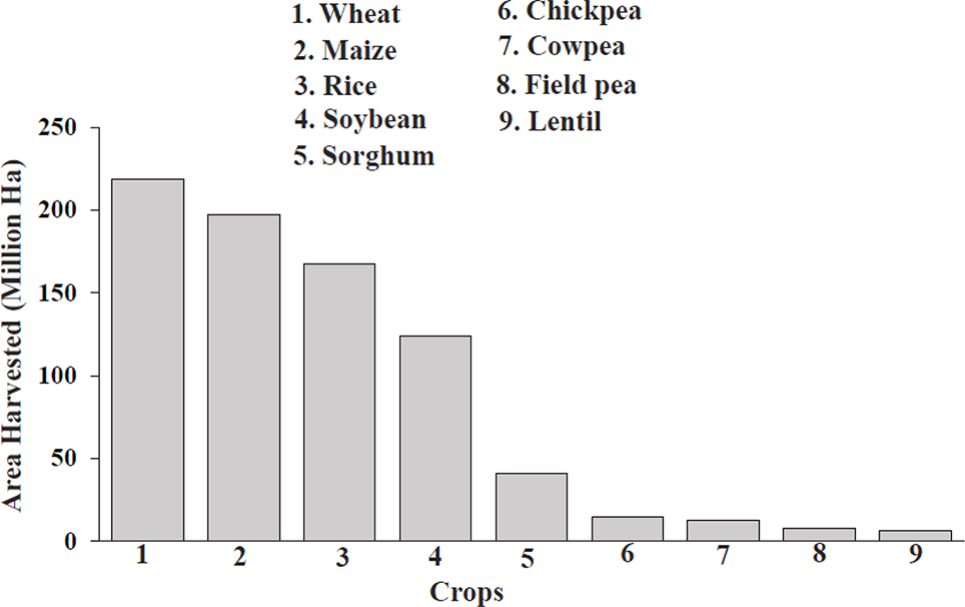
Comparison of area harvested for cereals and field pea in 2017 (FAOSTAT, 2019). The numbers above each bar represent the individual harvest totals of each crop.

In 2017, a total of 8,141,031 hectares of field pea were harvested globally (**Figure 1**), with the top producers consisting of Canada, Russia, China, India, and the United States (FAOSTAT, 2019); however, this is only a minimal fraction compared to cereal production. Cultivated land acreage for field pea and other pulses has been in steady decline over the past 30 years (Stagnari et al., 2017). Average yields have increased about 70–84% since 1974 for staple legumes, such as soybean, lentil, chickpea, and groundnut; in contrast, yields for field pea have increased but resulted in no net production gains due to decreasing land acreage (Foyer et al., 2016). The minimal expansion of pulses in agriculture is due to smaller and unpredictable yields, caused by susceptibility to environmental factors, and has resulted in a less-developed global market with decreased profits, disincentivizing farmers from using pulses for income while policymakers focus more attention and resources on cereals in developing countries (Foyer et al., 2016; Stagnari et al., 2017). These practices have compromised human nutrition, as cereals have less protein than field pea and pulse crops as well as inadequate levels of micronutrients, contributing to hidden hunger (Pingali, 2012). Pulses are also good sources of prebiotic carbohydrates (essential for gut health), fiber, minerals, vitamins, carotenoids, and polyphenols, allowing them to address health problems such as malnutrition, prenatal care, cardiovascular disease, diabetes, cancer, obesity, and gastrointestinal (GI)-related issues that plague both developing and developed nations (Welch, 2002; Foyer et al., 2016).

An additional hindrance to legume production is the high phosphorus requirement for nodule formation and function. Intensive mineral P fertilization has caused P surpluses in the soil of many countries, but deficits still exist in parts of Africa, the Northern U.S., South America, Eastern Europe, and Asia, likely due to multiple cycles of mono-crop farming or limited access to mineral fertilizers (MacDonald et al., 2011). Many resource-poor farmers practice subsistence agriculture, which utilizes organic principles such as no pesticides, chemical fertilizers, or industrial equipment. The soils they farm are generally poorer in nutrients, resulting in poorer yields and possibly poorer nutritional quality of the crop. For pulses such as field pea to be effective in combating hidden hunger, breeding efforts should be conducted to prepare varieties for these limiting environments. In addition, more specific breeding efforts should also focus on breeding field pea varieties solely for the organic environment to resolve the yield and nutritional discrepancies between conventional and organic agriculture.

### Organic Soil Vs. Conventional Soil

Organic agriculture is regarded as having healthier soils than conventional systems. Indeed, organic soil has higher soil organic matter, soil organic carbon, soil aggregate stability, and soil moisture content than conventional soils—all values that increase soil health and fertility (Schrama et al., 2018). Organic soils also have an increased level of biodiversity, as in a wider range of pollinators, insects, and earthworms, along with high microbial biomass and enzymatic activity (Hole et al., 2005). Despite healthier soils, limited herbicide and pesticide use along with additional weed pressure are limiting factors to productivity in organic agriculture. Additionally, N is a limiting nutrient in both conventional and organic production, but especially in organic systems that do not allow synthetic fertilizers as a source of N. Organic crops actually require twice as much N as conventional systems to achieve comparable yields (Seufert et al., 2012). Therefore, legumes are critical in organic systems, as they fix and efficiently use their own N, and supply it back to the soil from biomass after harvest at a rate of 40 million tons per year (Seufert et al., 2012; Udvardi and Poole, 2013).

Pulses face other nutrient constraints in organic agriculture due to their high P demand. Organic systems do not adequately replenish P supplies after harvest, leading to a deficit for the incoming crop (Oehl et al., 2002; Seufert et al., 2012). Additionally, sources of P for organic farming in the U.S. are restricted to FDA-approved manures and bone meal, as well as phosphate rock (Möller et al., 2018). For farmers that convert from conventional to organic management, decreases in available P in soils have been reported, meaning that the fertilizer is not adequate as a single source, and plants utilize P built up in the soil from previous fertilizer applications (Oehl et al., 2002). Overall, this means that organic agriculture is still dependent on nonrenewable sources of phosphorus, which decreases the sustainability of organic production.

One strategy to combat the negative impact of increased weed and disease pressure and nutrient limitations in organic environments is to identify breeding targets that fortify varieties to cope with these stressors. There are cereal organic breeding programs already underway (Wolfe et al., 2008; Jones et al., 2011). In field pea, genetic variation may also exist for phosphorus use efficiency (PUE), which would allow for the development of cultivars that are less dependent on P fertilizer input; this would benefit both conventional and organic growing systems. Additionally, PUE should be the main consideration for organic legumes, so that they can maintain nitrogen-fixing activity, yield stability, and adequate biomass under phosphorus-deficient conditions (van de Wiel et al., 2016). This will also prolong the period residual P can contribute to production, allowing it to be used more efficiently (van de Wiel et al., 2016). PUE is a complex trait, involving multiple pathways and gene networks, but can be broken into the ability of the plant roots to acquire P from the soil and the plant’s ability to remobilize and allocate P to sustain productivity (van de Wiel et al., 2016). Unfortunately for field pea, there is a dearth of genomic information and resources regarding these processes, so more research should be conducted to identify these genomic regions as field pea becomes more popular in the health food market.

### Phosphorus Physiology of Legumes

**Figure 2:**
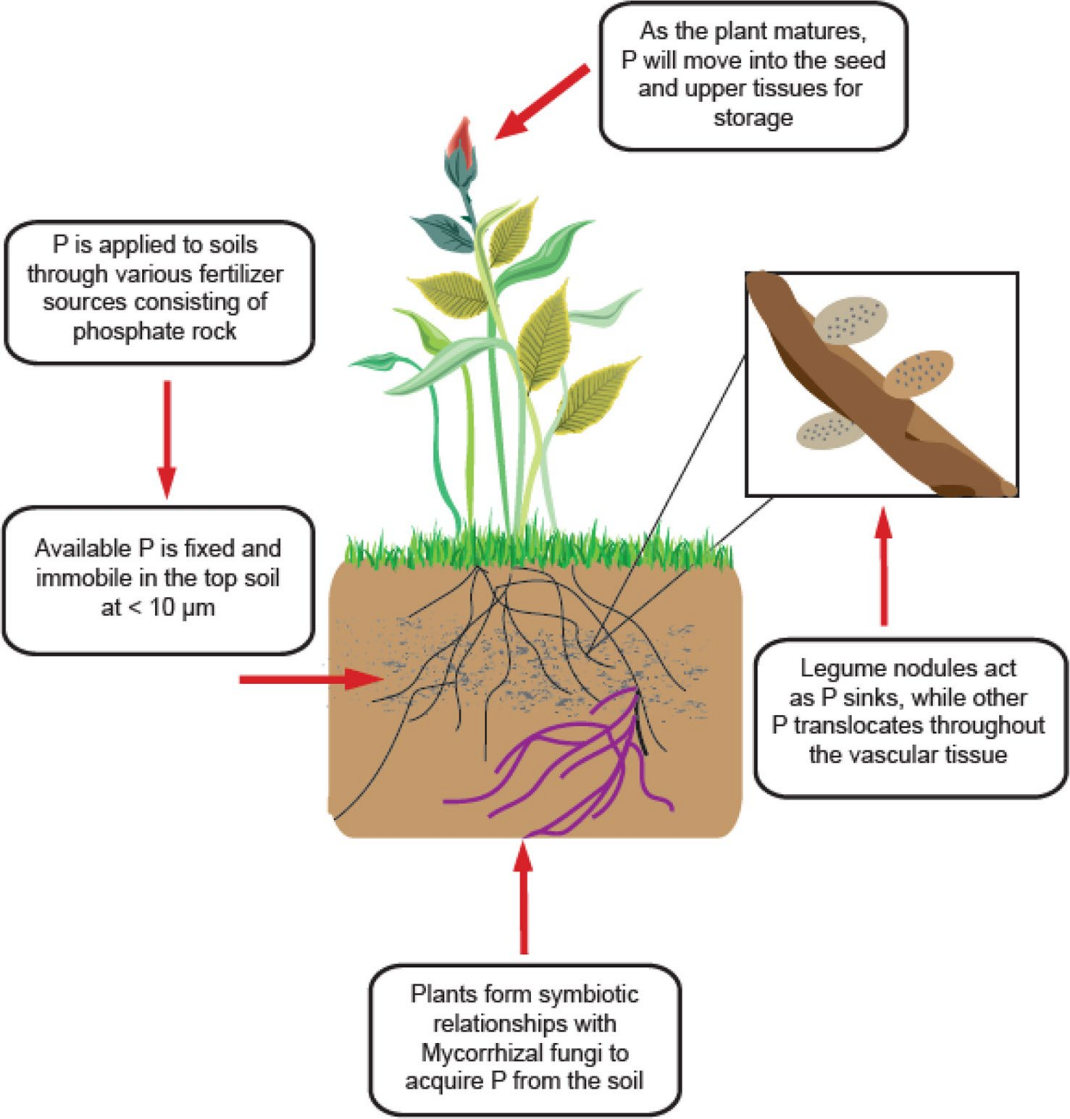
General scheme of P acquisition and utilization of legume plants.

Phosphorus is only available to plants in its inorganic forms (Pi) as H_2_PO_4_^−^ and HPO_4_^2–^ which exist in very small concentrations in the soil (< 10 µm) (**Figure 2**) (Schachtman et al., 1998). Availability is also highly dependent on soil pH, as P forms insoluble complexes with Al and Fe under acidic conditions and Ca under alkaline conditions (Seufert et al., 2012). This presents an issue for all forms of agriculture worldwide, as most soils are acidic (Reganold and Wachter, 2016; Slessarev et al., 2016). All plants have adopted mechanisms to combat the unavailable nature of P, such as altered root architecture, organic acid exudation, specialized transport systems, lipid remodelling, and symbiosis with arbuscular mycorrhizal fungi (AMF) (**Figure 2**) (Vance et al., 2003; Oehl et al., 2004). AMF are especially important in organic systems, where less is P available, and plants rely more heavily on these fungi to gather and supply P and other nutrients (Oehl et al., 2004). P is applied to soils from phosphate rock sources, and becomes immobile in the soil, with small concentrations accessible to the roots. Plants will form symbiotic relationships with Mycorrhizal fungi for greater P acquisition. For legumes specifically, high concentrations of P exist in the nodules to maintain nitrogen-fixing function. Due to stress or senescence, P is remobilized from younger tissues and moves into upper leaves and seeds for storage as phytic acid.

P uptake is regulated by high and low affinity transporters located throughout the vascular system of the plant. The root hair mediates the uptake of Pi from the soil, where it is transported across the root plasma membrane by a P-type H+-ATPase pump (Vance et al., 2003). From there, the Pi is transported into the nodules or upward into the shoot by the xylem where it goes to individual cells (Vance et al., 2003). The cytoplasm maintains a strict Pi concentration of around 5–10 mM (Schachtman et al., 1998); if no deficiency is detected, the Pi will be stored in the vacuole until P stress signals are detected or senescence begins. If Pi becomes limited throughout the plant, vacuolar Pi will efflux into the cell cytoplasm and be allocated to other vital tissues, such as legume nodules. Additionally, during senescence Pi is again effluxed from the vacuole, where it is transported from older leaves to younger leaves and seeds by the xylem and various transporters (Robinson et al., 2012; Yang et al., 2017; Xu et al., 2019). Once Pi reaches the seed, it is stored as phytic acid or phytate and utilized during seed germination to establish enough growth until the seedling can take up nutrients on its own (Robinson et al., 2012; Yang et al., 2017; Xu et al., 2019). Some crops have more phytic acid than others, with field pea containing a high amount. Phytic acid is an antinutrient, meaning it binds to other minerals and decreases bioavailability, making crops high in phytic acid undesirable for animal and human consumption.

Adaptations for P limitation in legumes specifically involve preferential allocation of P to nodules to maintain N fixation, rhizosphere acidification through root exudation, formation of proteoid roots, and modified carbon metabolism and mycorrhizae formation to control for competition with nodule development (Vance et al., 2003). Therefore, legumes will suffer greatly if P is limited, as they will be unable to maintain nodule function and overall productivity due to decreased photosynthetic ability, tissue expansion, and flower formation (Sa and Israel, 1991; Vance et al., 2003; Sulieman and Tran, 2015). Most research on these processes has been conducted in soybean (*Glycine max*), so there is a need to investigate responses in field pea specifically. There is also a gap in the literature with respect to specific links between phosphorus deficiency and nutrient bioavailability. The nutritional value of organic crops compared to conventional crops is an additional gray area; however, it can be inferred that limited P not only affects human health through reduced yield, but could also decrease protein, carbohydrate, and lipid content due to P’s involvement in plant metabolic activities.

### P Efficiency and Plant-Soil-Microbe Interactions

As previously discussed, most vascular land plants have formed evolutionary beneficial relationships with AMF, but increasing evidence indicates the entire soil microbiome is a mediator for plant health. This relationship is caused by the secretion of photosynthates and carbon sources into the rhizosphere, acting as a tradeoff for various microbes (Bakker et al., 2018), which then provide the plant with various health benefits such as nutrient availability. The composition of soil microbiomes is largely dependent on the soil type and the environment, but plant genotype can also influence microbial populations depending on the type of root exudate and hormones it produces. For example, Arabidopsis accessions differ in their ability to colonize *Pseudomonas* bacteria, leading to some accessions being more disease resistant than others (Bakker et al., 2018). Additionally, salicylic acid exudation by Arabidopsis influences the composition of root microbiomes, again demonstrating the plant has some influence over the rhizosphere (Bakker et al., 2018).

The soil microbiome is implicated in P acquisition, and AMF and rhizobia interactions with legumes are well characterized. Legumes exude flavonoids into the rhizosphere that attract rhizobia to stimulate nodule formation as well as allow for AMF interaction; both lead to enhanced P availability for the legume (Jacoby et al., 2017). Another mechanism is the modification of root exudates under P limitation. Maize and rice alter their exudates to contain more carbohydrates and sugars to provide an energy source for AMF formation (Carvalhais et al., 2011). Increased sugar exudation has also been identified in *Pisum sativum*, which then increased the mineralization of insoluble P by microbial activity (Schilling et al., 1998).

A result of conventional farming is the notion that breeders have inadvertently selected for traits that weaken plant-microbe interactions due to intensive fertilizer and pesticide use (Bakker et al., 2018). Studies in barley, maize, and Arabidopsis indicate differences in rhizospheres between wild and domesticated material as well as natural variation among accessions (Bakker et al., 2018). More studies must be done to dissect the contribution of genetic variability to microbial communities, as these could be targets for organic plant breeding initiatives. The lack of efficient plant-soil-microbe interactions in conventionally bred crops could help explain reduced yields when these varieties are introduced to organic environments, where a stronger soil microbiome relationship would be beneficial due to the lack of fertilizers and pesticides (Jones et al., 2011; Bakker et al., 2018). Organic pulse breeding should focus on microbial interactions to improve P acquisition, and genomic studies should be performed in the diverse germplasm to discover any beneficial traits that may have been lost from modern day varieties over time.

### Field Pea and Phosphorus Use Efficiency

PUE is defined as the total biomass per unit of P taken up and encompasses the plant’s ability to acquire P from the soil then translocate, remobilize, and efficiently utilize it for various physiological processes (Shenoy and Kalagudi, 2005; Veneklaas et al., 2012). The overall goal of PUE breeding is to determine genomic regions that contribute to these processes and allow crops to grow and yield at optimal levels under low P conditions. Generally, a greater focus has been placed on improving P acquisition from the soil by identifying genes and processes associated with root systems architecture and rhizosphere modifications under P deficiency through quantitative trait loci (QTL), genome-wide association study (GWAS), and biotechnological methods (Rose and Wissuwa, 2012; Veneklaas et al., 2012; van de Wiel et al., 2016). While these aims would allow crops to scavenge residual P built up in the soil from the over-application of fertilizer, this strategy may deplete P from nutrient-poor soils and further upset the balance of fertilizer input to uptake, again leading to P depletion (van de Wiel et al., 2016). Remobilization of P from vegetative tissues is the main source of P for reproductive tissues, impacting yield and seed quality, so understanding and improving allocation efficiency is a necessary goal for crops with better PUE. Additionally, increased P acquisition for field pea may lead to a greater accumulation of phytate in seeds, thus decreasing the bioavailability of nutrients upon consumption. Therefore, in terms of field pea, a balance must be achieved between P acquisition and internal P utilization to avoid excess accumulation of phytate.

Several studies in grain crops have concerned PUE (Rose and Wissuwa, 2012), but none, as far as we are aware, have been conducted in field pea. Genetic variation is visible among pulse crop varieties, so field pea studies should not be ignored. A recent study in chickpea (*Cicer arietinum L*.) showed great variation among diverse germplasm and commercial varieties for various aspects of PUE, such as total biomass, photosynthetic rate, root structure, and root acquisition under limited P conditions (Pang et al., 2018). Several accessions from the diverse germplasm were shown to outperform commercial chickpea varieties in terms of these criteria, indicating genetic variation that may be exploited for PUE breeding purposes (Pang et al., 2018).

Another challenge for PUE in organic agriculture is that most studies are conducted in greenhouses or under conventional management, which differs significantly from organic practices (Rose and Wissuwa, 2012). Therefore, more PUE studies should be conducted with the field and growing environment in mind to generate more realistic results. Studies for PUE in field pea and other pulses should be increased in general and can be aided by recent genotypic data for the diverse field pea germplasm (Holdsworth et al., 2017). Breeding for PUE will significantly benefit organic agriculture as it is a P-limited environment, where the ratio of P input to P uptake is already off-balance and inadequate. Research concerning field pea PUE should be prioritized in biofortification programs, as adequate phosphorus utilization will aid in increasing the amount and bioavailability of nutrients.

### Biofortification Potential of Legumes

As previously stated, agriculture not only faces the issue of yield deficits for a growing population but also increased incidences of hidden hunger as more people develop micronutrient deficiencies. A potential solution to overcome micronutrient deficiencies is to increase consumption of pulses, which contain superior protein, carbohydrate, fiber, and micronutrient content compared to cereals, as well as complementary amino acid profiles to those found in cereals (Rehman et al., 2018). Field pea is also attracting positive attention in health food markets, as they are rich in protein (23.5 g protein per 100 g) and a viable substitution to wheat and egg-based products. Protein extraction is reported to be most successful from field pea, and the protein structure of peas is the most similar to egg and stabilizes snacks and cereals most similarly to gluten when compared to other alternative protein sources. By increasing protein content of field pea, a more significant profit and expansion of the field pea market may take place, paving the way for more initiatives to support growers of field pea and other pulse crops. Another solution is to boost biofortification breeding efforts to increase nutritional value where legumes lack to supplement a low diversity diet due to climate change and crop availability.

However, an issue relating both biofortification and phosphorus use efficiency is the conversion of P to *myo*-inositol-1,2,3,4,5,6-hexa*kis*phosphate (InsP6), also known as phytic acid, which acts as an antinutritional factor by decreasing the bioavailability of nutrients in pulses when consumed (Rehman et al., 2018) (Raboy, 2003). As P is taken up by the roots from the soil, it is converted to glucose 6-phosphate (G6P) before entering the inositol phosphate pathway through the conversion of G6P to inositol 3-phosphate (Ins3P) by myo-inositol(3)P1 synthase (MIPS) (Raboy, 2003). From there, every carbon of the 6-carbon ring is phosphorylated until it becomes InsP6 or phytic acid (Raboy, 2003). Phytic acid is the primary storage form of P in seed and is often bound in phytate salts to Ca or Fe (Raboy, 2003). These structures cannot be broken down by humans as they lack the necessary enzymes (Raboy, 2003). As P is found throughout the plant and stored in various tissues during vegetative and reproductive growth, biofortification efforts should aim to understand the mobilization of P throughout the growing cycle. Additionally, more research should be dedicated to the speciation of P within the plant to identify genetic variation for P conversion and phytic acid content. These are concerns of both PUE and biofortification research as plants must efficiently take up P for growth, as well as convert P selectively for nutrient availability.

Several low phytic acid varieties have been developed in wheat, maize, barley, rice, and soybean through transgenic and biotechnological methods (Wilcox et al., 2000; Larson et al., 2000; Raboy et al., 2000; Guttieri et al., 2004; Rasmussen and Hatzack, 2004). Warkentin et al. developed low phytate field pea variety CDC Bronco *via* EMS to produce the desired mutation in MIPS to halt conversion to higher inositol phosphate molecules. Overall grain phytic acid is reduced, but there are reports of several agronomic issues, such as decreased stress tolerance, germination, growth, and seed weight, as the plant cannot store enough P to use during vegetative processes, in addition to reports of reduced protein content in winter wheat (Raboy et al., 1991; Oltmans et al., 2005; Bregitzer and Raboy, 2006; Warkentin et al., 2012; Rehman et al., 2018). While requiring less P overall, these lines are often stunted, with lower biomass and yield compared to commercial varieties, further illustrating the problem of less P uptake vs. high productivity (Raboy, 2009; Warkentin et al., 2012; Sparvoli and Cominelli, 2015). Additionally, for organic systems, transgenic and chemical mutants are not currently allowed, so they have no use in sustainable agriculture. Furthermore, transgenics and chemically modified seeds are not allowed in the food market, may be banned as in the EU, and consumer approval is generally considered negative or unclear (Lucht, 2015). A more conventional plant breeding approach could be more successful in terms of developing varieties with reduced phytic acid accumulation and positively impact biofortification.

### Future Directions

Field pea is highly nutritious and beneficial to agriculture systems, along with other pulses. However field pea is especially advantageous in terms of protein content and extractability. This is the powerful avenue to expand field pea production as consumer interest in health foods, and meatless alternatives grow. Field pea could be biofortified for protein as well as micronutrient content, to increase marketability, as well as ability to fight hidden hunger. The adoption of organic principles is necessary, and organic agriculture should expand, as nonrenewable resources like P begin to deplete. Field pea and other pulses are critical to sustainable agriculture but will suffer from soil P deficiencies, affecting their beneficial status in organic systems and negatively affecting crops that depend on their nitrogen-fixing capabilities.

To adequately prepare and avoid the negative impacts of phosphorus deficiency, a thorough investigation of the genetic diversity of field pea in terms of phosphorus use efficiency is necessary. We hypothesize that there will be variation in the ability of different field pea accessions to acquire, mobilize, and store P under P deficient conditions. Phenotyping could reveal superior yield, nutritional value, and other important agronomic characteristics of some accessions and physiological and genetic analyses would aid in elucidating the biological mechanism. It is possible that there are accessions containing genes that can be incorporated into elite breeding lines to confer benefit in P deficient environments. The investigation regarding natural genetic variation within the germplasm for differing rates of P speciation should also be considered. For example, one genotype may preferentially convert to Ins3P or other lower inositol phosphates over phytic acid, leading to increased Pi and nutrient bioavailability in the seed, and allowing for low phytic acid lines to be conventionally bred rather than mutagenized. This would increase field pea production sustainability and allow new varieties to be developed for consumer use. In terms of biofortification, identifying field pea genotypes with higher potential for micronutrient accumulation, especially under P and nutrient-deficient environments, will be critical, through the understanding of variation in acquisition and translocation of nutrients to the seed (Welch and Graham, 2004). The common bean core collection has demonstrated variation in Fe and Zn uptake, as well as elucidated a negative correlation between the two during breeding efforts, so these antagonistic relationships must also be discovered and considered (Welch and Graham, 2004).

Despite growing interest in field pea, at the time of this review, there is still no reference genome published, and when one is released, it will always be the first assembly, meaning it will likely need to undergo revisions as technology and genomic understanding of field pea and improve. A single core collection consisting of 431 pea accessions does exist and shows ample genetic variation between accessions, allowing for more genetic studies ((Holdsworth et al., 2017). By using GWAS and other omics methods, questions concerning organic and nutritional breeding may be answered. Additionally, more funding for field pea and pulse research is required, as it has been limited by unstable yields and forgotten by institutions, leading to little germplasm improvement. Government agencies must get involved to promote awareness and create funding opportunities to improve field pea and pulse germplasm, so that legume profitability may increase to better compete with cereals. This is a key measure to ensure people have access to a diverse nutritional diet to combat hidden hunger.

## Conclusion

A primary focus of agriculture should be to increase sustainability and nutritional value to the human diet through the adoption of more organic practices; this includes diversification of staple crops to include more pulses such as field pea and decreased dependence on nonrenewable resources such as phosphorus. For these goals to be met, more organic-specific breeding initiatives should be undertaken, and more research should be conducted on field pea. The dearth of knowledge on pulses compared to cereals is detrimental to agricultural research and the human diet, so more genomic studies should be conducted to increase productivity and adoption of pulses. Research concerning PUE will benefit farmers and consumers of all types by decreasing reliance on fertilizers and maximizing productivity for already P-deficient soils. Because field pea is consumed globally by both developed and at-risk populations, these efforts could help increase global health overall and combat hidden hunger.

## Author Contributions

SP: drafted the manuscript and the doctoral student working on the project. DT: edited the draft and the project PI.

## Conflict of Interest

The authors declare that the research was conducted in the absence of any commercial or financial relationships that could be construed as a potential conflict of interest.
